# Exploring the role of drug-induced sleep endoscopy in optimizing upper airway stimulation therapy for non-responders

**DOI:** 10.1007/s11325-025-03468-z

**Published:** 2025-09-13

**Authors:** E. Kant, J. A. Hardeman, R. J. Stokroos, M. P. Copper

**Affiliations:** 1 Department of Otorhinolaryngology Head and Neck Surgery, Sint Antonius Hospital, Nieuwegein, the Netherlands; 2 Department of Otorhinolaryngology, Head and Neck Surgery, University Medical Center Utrecht, Heidelberglaan 100, 3584 CX Utrecht, the Netherlands; 3 University Medical Center Utrecht Brain Center, University Medical Center Utrecht, Utrecht, the Netherlands; 4 Department of Pulmonology, Sint Antonius Hospital, Nieuwegein, the Netherlands

**Keywords:** Drug-induced sleep endoscopy, DISE, Upper airway stimulation, Hypoglossal nerve stimulation, Titration

## Abstract

**Purpose:**

Upper airway stimulation by means of unilateral hypoglossal nerve stimulation is an alternative therapy for patients with obstructive sleep apnea and continuous positive airway pressure intolerance or failure. Optimal device settings are essential for effective treatment of obstructive sleep apnea with this therapy. Some patients experience therapy failure, despite standard titration methods. We perform a drug induced sleep endoscopy with upper airway stimulation in these patients. This study provides an overview of this procedure and our patient outcomes.

**Methods:**

Retrospective chart review of patients implanted with an upper airway stimulation device between 2015 and 2022.

**Results:**

The drug-induced sleep endoscopy with upper airway stimulation therapy is performed in 29 non-responders of therapy. In seven patients, a change in device settings was sufficient to prevent an upper airway collapse during the drug-induced sleep endoscopy, while additional therapy was recommended for the remaining. Eventually, six patients were fitted with a change in device settings and fourteen patients were treated with additional therapy, being oral appliance therapy, barbed wire pharyngoplasty or alternative upper airway surgery. At follow-up, the apnea–hypopnea index declined significantly from 41.6 ± 15.0 to 23.2 ± 9.9 events/hour and resulted in eight additional responders to therapy.

**Conclusion:**

The drug-induced sleep endoscopy might be a valuable addition in the clinical pathway of upper airway stimulation therapy. It provides a better evaluation of the stimulation process and offers the opportunity for alterations in settings or targeted additional therapy.

## Introduction

The Upper Airway Stimulation (UAS) system is an implantable device used as an alternative therapy for patients with moderate-to-severe obstructive sleep apnea (OSA) and continuous positive airway pressure (CPAP) intolerance or failure. The system functions by detecting the individual’s breathing pattern and accordingly stimulating the protrusor fibres of the hypoglossal nerve (n. XII), resulting in tongue protrusion. Due to palatoglossal coupling, obstruction at the palatal level is simultaneously relieved. As a result, a patent upper airway is obtained, preventing obstructive apneas to occur [1, 2].

Optimal device settings play an important role in gaining patency of the upper airway. Therefore, adequate device settings, balancing effectiveness and comfort, are crucial [3]. The UAS device will be activated using standard settings approximately one month post-implantation. Patients are instructed to increase the stimulation amplitude at home. After three months, a titration polysomnography (PSG) will be performed, in which device settings can be optimized during natural sleep [3]. Subsequently, an awake titration, adjusting settings while visualizing the effect on the upper airway using a flexible laryngoscope, can be performed in patients with insufficient response to initial settings after the titration PSG.

Most patients experience beneficial effects of the UAS therapy after these titration steps. However, a minority still lacks adequate improvement. We perform a drug-induced sleep endoscopy (DISE) with UAS therapy turned on in these non-responders of UAS therapy. In this article, we aim to provide an insight in our working process, by presenting an overview of this procedure and our results of this potential additional step in the clinical pathway of UAS.

## Methods

### Study design and study population

This exploratory study included patients implanted with an UAS device (Inspire® Medical Systems, Inc.) in the period between 2015 and 2022 and who underwent a DISE with UAS therapy. Patient data was retrospectively collected and managed using REDCap electronic data capture tools hosted at the Sint Antonius Hospital [4, 5]. Age, gender, body mass index (BMI), baseline and follow-up sleep study outcomes, pre-operative DISE phenotypes, records of a DISE with UAS therapy, additional treatment and treatment effects were collected. Sleep study outcomes were obtained from an in-laboratory PSG or polygraphy (PG).

### DISE with UAS

The DISE is carried out in a quiet operating room with dimmed lights with the patient lying in a supine position. All procedures are captured on video and available for later review. A bolus of propofol is administered, followed by manual titration to maintain sedation. The targeted sedation depth is the presence of snoring and/or apneas. Bispectral Index was not used during the DISE. The upper airway is assessed using a flexible endoscope and scored according to the VOTE-classification [6] with and without UAS stimulation.

**Fig. 1:**
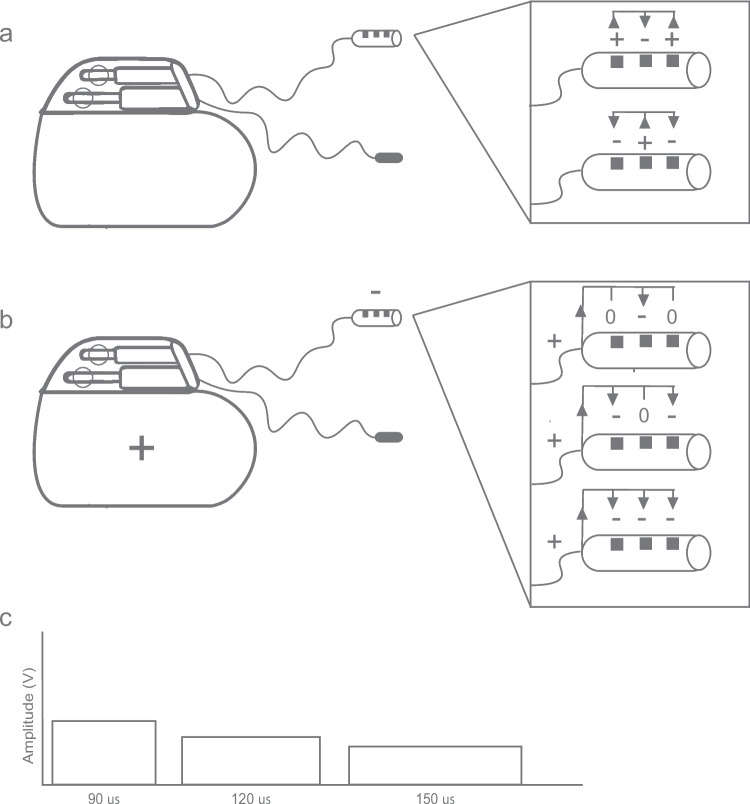
Schematic illustration of the mechanism of the implantable upper airway device (Inspire® Medical Systems, Inc.), showing the three components and various settings for hypoglossal nerve stimulation. **a**. The schematic representation of the three components of the device; (i) the implantable pulse generator, (ii) the sense lead, which detects the breathing pattern via a pressure sensor, (iii) the stimulation lead, which stimulates the hypoglossal nerve. It depicts a scenario with a bipolar electrode configuration [+ - +] or [- + -], where current flows between the three electrodes within the cuff. **b**. The schematic shows the same setup with a monopolar configuration. [0–0], where the middle electrode serves as the cathode, with the outer electrodes inactive and the implantable pulse generator acting as anode; [−0-], where the outer electrodes function as cathode, the middle electrode is inactive and the implantable pulse generator acts as anode; and [---], where all three electrodes act as cathodes, with the implantable pulse generator as the anode. **c.** Stimulation of the nerve is contingent upon the stimulation amplitude, the impulse strength in voltage, and the pulse width, duration of pulse administration in microseconds. Longer pulse widths permit lower amplitudes to achieve equivalent overall stimulation, making these three blocks comparable in stimulation of the hypoglossal nerve

After a first assessment, the effect of different device settings on the upper airway is examined. Several parameters of the UAS device settings exist. The cuff electrode of the stimulation lead consists of three stimulation electrodes, resulting in the opportunity to alter the pattern of electrostimulation of the hypoglossal nerve. Most of the adjustments during a DISE are related to this setting, the electrode configuration. Additionally, the other settings can be adapted as required. These settings are the stimulation amplitude, the impulse strength in voltage (V), the pulse width, the time a pulse is administered (µs) and pulse rate, the frequency (Hz) of the pulse [7] (Fig. 1). The default settings are a bipolar configuration [+ - +], a pulse width of 90 µsec and a frequency of 33 Hz.

First, the incoming settings are evaluated. Patients typically arrive with the default bipolar electrode configuration setting [+ - +], in which current flows within the cuff between the three electrodes. Another bipolar [- + -] setting exists, and can be tried to evaluate its effect, but is not very likely to give a different airway response than the default settings. The main alternative electrode configuration settings are the monopolar settings, in which the current moves between the cuff electrode and the implantable pulse generator (IPG), creating a different response on the upper airway. To start, the [0–0] configuration is attempted, in which the middle electrode serves as the cathode, the outer electrodes are in off state, and the IPG acts as anode. If insufficient, [−0-], in which the outer electrodes act as the cathode, and [---], using all three electrodes as cathodes, are used. The monopolar setting is higher in intensity and may cause more sleep arousal. Additionally, the more electrodes are activated in a monopolar setting, the higher the intensity of the current is. Therefore, the stimulation amplitude should be reduced to roughly half of the incoming bipolar setting in [0–0] and [−0-] and one third in [---] [7, 8]. The stimulation amplitude can be titrated up in all electrode configurations with steps of 0.2 V to evaluate effect.

Secondly, altering the comfort settings by adjusting the pulse width and frequency is possible and can be interesting due to the possibility of reducing the stimulation amplitude [9, 10], though challenging to evaluate due to the inability of communication with the patient. Therefore, this task is typically completed during awake titration.

At the end of the procedure, the device is changed to the optimal settings. The upper airway responding to the most optimal device settings is described using the VOTE classification. Patients gain the possibility to titrate stimulation amplitude at home. If altering device settings does not result in a satisfactory upper airway patency, additional therapeutic recommendations are provided. Additional treatment options are either oral appliance therapy (OAT), a barbed wire reposition pharyngoplasty (BRP) or alternative upper airway surgery. The choice between these additional treatment options is based on the findings during DISE. OAT is selected when a jaw thrust together with optimal settings of the UAS opened the airway adequately. BRP is chosen in cases of isolated palatal collapse, while other surgical interventions are considered when the obstruction is localized to those structures and other levels of the upper airway are resolved well with optimal device settings of the UAS.

Patients are contacted after two weeks to assess comfort and efficacy. A follow-up PSG or PG is provided after approximately three months post-intervention for surgical cases. For patients undergoing device setting adjustments or advised to use OAT, the evaluation occurs once optimal settings are achieved.

### Statistics

IBM SPSS Statistics (Version 26, New York, USA) was used for statistical analyses. Prism GraphPad (Version 9.3.1, San Diego, USA) was used for graphical presentation. Continuous variables were reported as means and standard deviations (SD) and categorical variables as numbers and frequencies (percentages). Comparisons between or within groups were performed using Student’s t-test, Mann–Whitney *U* test, χ^2^ -test and Fisher’s exact test. A two-sided p-value < 0.05 was considered statistically significant.

## Results

A DISE with UAS therapy was performed in a subgroup of 29 patients from a total of 114 patients who underwent UAS implantation during our study period. These 29 patients underwent DISE with UAS therapy because they were non-responders to treatment. This subgroup consisted predominantly of males (*n* = 28, 97%), with a mean age at implantation of 57.3 ± 9.4 years, a mean BMI of 27.9 ± 2.7 kg/m^2^ and a pre-operative apnea–hypopnea index (AHI) of 37.4 ± 7.6 events/hour (Table 1). These patients had a follow-up AHI of 39.0 ± 13.4 events/hour, which was measured before the DISE with UAS. All baseline characteristics, including respiratory parameters and the subjective questionnaires were comparable between this subgroup and all implanted patients. The baseline upper airway collapse patterns of these 29 patients are described in Table 2, which were similar compared to the upper airway collapse patterns of all patients implanted in the study period. Since comparison with the overall implanted patients revealed no significant differences, no preoperative factors were identified that could predict non-response. The following results focus exclusively on this subgroup of 29 patients.

**Table 1:** Baseline characteristics

	Patients (*n* = 29)
Male	28 (97%)
Age (years)	57.3 (9.4)
BMI (kg/m2)	27.9 (2.7)
Previous tonsillectomy	16 (55%)
Previous OSA-surgery^£^	8 (28%)
Pre-implantation AHI (e/h)	37.4 (7.6)
Pre-implantation ODI ≥ 4% (e/h)	26.9 (10.9)

Data described before implantation of the upper airway stimulation device and is presented as n (%) or mean (SD). BMI = body mass index, OSA = obstructive sleep apnea, AHI = apnea–hypopnea index, ODI = oxygen desaturation index, e/h = events/hour, £ other than tonsillectomy, being palatal surgery (*n* = 6), turbinate surgery (*n* = 1), multilevel surgery (*n* = 1)

**Table 2:** The VOTE classification during drug-induced sleep endoscopy pre-implantation

*n* = 29	Pattern								
Level	Anteroposterior			Lateral			Concentric		
	None	Partial	Complete	None	Partial	Complete	None	Partial	Complete
Velum	1 (3%)	2 (7%)	26 (90%)						
Oropharynx				22 (76%)	4 (14%)	2 (7%)			1 (3%)
Tongue base	7 (24%)	12 (41%)	10 (35%)						
Epiglottis	3 (10%)	10 (35%)	13 (45%)		3 (10%)				

In 25 patients the collapse patterns with optimal settings of the UAS were recorded and described according to the VOTE classification system. Twelve of these patients exhibited a persistent unilevel complete anterior–posterior velar collapse, three patients had a unilevel complete concentric velar collapse, four patients had a complete collapse only at the level of tongue base and/or epiglottis and two patients had multilevel collapse. The remaining had no upper airway collapse with optimal settings (Table 3).

**Table 3:** The VOTE classification during drug-induced sleep endoscopy with optimal stimulation settings of the upper airway stimulation system

*n* = 25	Pattern								
Level	Anteroposterior			Lateral			Concentric		
	None	Partial	Complete	None	Partial	Complete	None	Partial	Complete
Velum	6 (24%)	4 (16%)	12 (48%)						3 (12%)
Oropharynx				24 (96%)	1 (4%)				
Tongue base	19 (76%)	3 (12%)	3 (12%)						
Epiglottis	18 (72%)	1 (4%)	5 (20%)			1 (4%)			

In total, sixteen patients had a lack of palatoglossal coupling, despite optimal UAS settings. These patients were comparable to the entire cohort and all patients who underwent a DISE, in terms of baseline patient characteristics, pre-implant respiratory parameters and pre-implant DISE phenotypes.

In seven patients, a change in device settings was sufficient to prevent upper airway collapse during the DISE. One patient did not tolerate these new device settings, resulting in six patients with new device settings, being a change in electrode configurations (*n* = 5, all to a monopolar setting) or pulse width (*n* = 1, to 120 μsec).

**Fig. 2:**
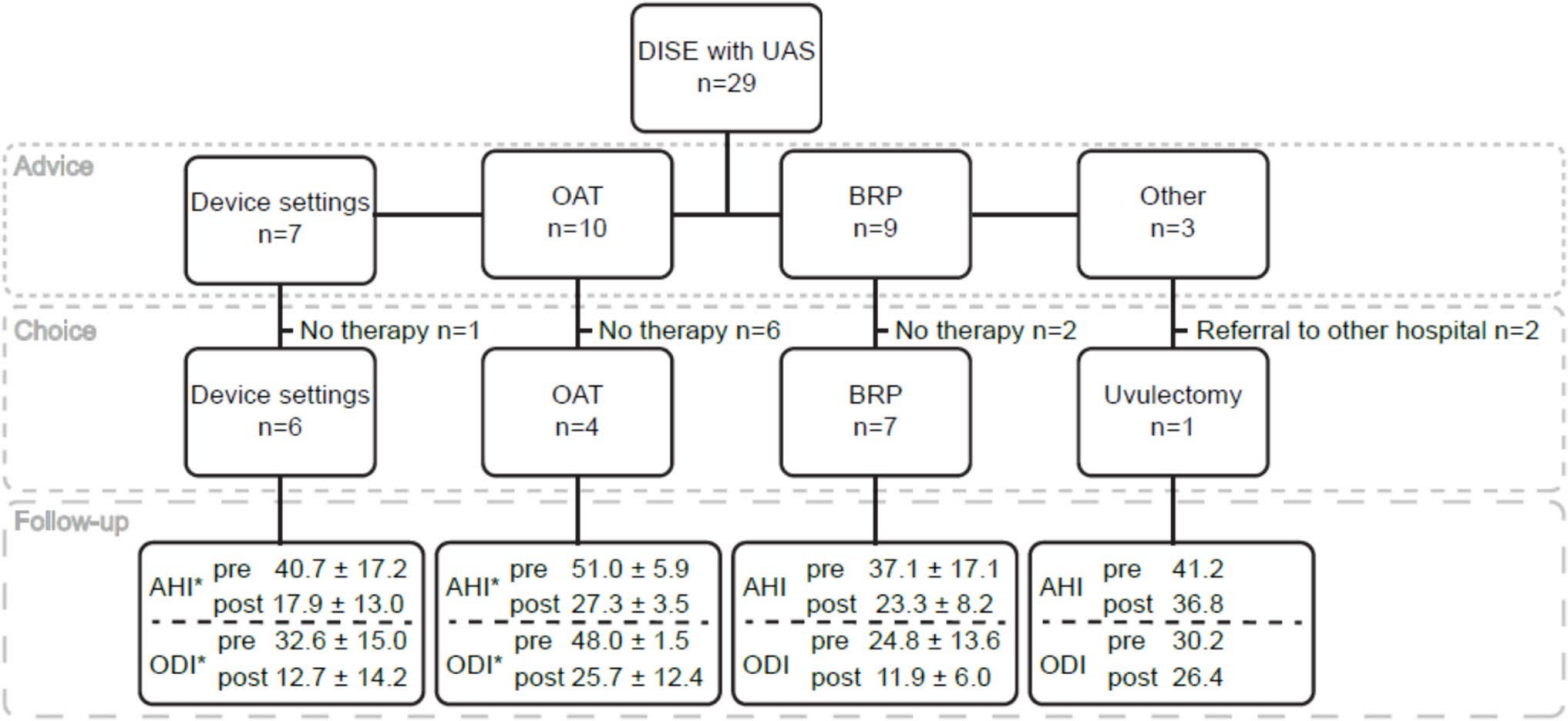
A chart illustrating the pathway after drug-induced sleep endoscopy with upper airway stimulation, outlining the advice provided by the surgeon, the patients’ decision, and the subsequent follow-up following the chosen treatment. Numbers in mean ± SD events/hour, values before procedure and afterwards (e.g. with (additional) treatment or change in device settings). UAS = upper airway stimulation, DISE = drug-induced sleep endoscopy, OAT = oral appliance therapy, BRP = barbed wire reposition pharyngoplasty, AHI = apnea–hypopnea index, ODI = oxygen desaturation index ≥ 4%, statistics by a paired student t-test, * *p* < 0.05

For the remaining patients, adjusting device settings proved to be insufficient. For these patients, additional treatment options were recommended, including OAT (*n* = 10), BRP (*n* = 9) or alternative upper airway surgery such as uvulectomy, trans oral robotic surgery and maxillo-mandibular advancement. The latter two patients were referred to another center. Twenty patients choose the suggested type of therapy (device settings *n* = 6, OAT *n* = 4, BRP *n* = 7, alternative upper airway surgery (*n* = 3)) and eighteen had a follow-up in our hospital (Fig. 2). Reasons for decline of therapy were device intolerance (*n* = 1), OAT-intolerance after used as additional therapy (*n* = 3), the wish for no further treatment (*n* = 4) or the return to CPAP therapy (*n* = 1). There was no difference in pre-DISE AHI or oxygen desaturation index (ODI) ≥ 4% between patients opted for therapy and those who did not (AHI: CI 95% (−17.3, 4.6) *p* = 0.245, ODI ≥ 4%: CI 95% (−15.9, 6.7), *p* = 0.155).

Among the eighteen patients who had a follow-up after approximately three months after additional therapy or device setting modifications, the average AHI decreased by 18.4 ± 15.9 events/hour (from 41.6 ± 15.0 to 23.2 ± 9.9 events/hour, 95% CI (10.6, 26.3), *p* < 0.001*)*. The ODI ≥ 4% declined 16.5 ± 14.0 events/hour (from 32.4 ± 14.4 to 15.9 ± 11.5 events/hour, 95% CI (8.8, 24.2), *p* < 0.001*)*) (Fig. 2). This resulted in eight more patients being classified as treatment responders according to the Sher’s success criteria (decrease ≥ 50%, AHI < 20 e/h). These were five out of the six patients who had a change in device settings, three out of the seven patients with a BRP and none of the five patients with OAT or uvulectomy. A patient-reported outcome, by means of the Epworth Sleepiness Scale, was collected in eleven of the patients and changed from 10.3 ± 5.3 to 8.8 ± 5.0 (95% CI (−0.2, 3.1), *p* = 0.083).

## Discussion

The efficacy of UAS treatment can be significantly improved through a carefully conducted follow-up trajectory [3]. The DISE with UAS may serve as a valuable additional intervention in this clinical pathway of UAS therapy, particularly when other titration methods have failed. We recommend considering it as a final step after exhausting alternative titration methods. This low-complexity procedure can provide a deeper understanding of the issue during stimulation and allows for assessment of the need for additional therapy in these patients.

UAS treatment response varies among patients, with some remaining non-responders. The reasons for reduced therapy effectiveness are not always clear. Ensuring the opening of both the airway at the tongue base and the palatal level is crucial for effective treatment. Previous studies emphasize the significance of palatal coupling, highlighting it as one of the most important factors in non-responsiveness to therapy [2, 11, 12]. However, the exact reasons for the absence of palatal coupling, particularly in patients considered viable candidates, remain unclear. In our cohort, the lack of palatal coupling was the most common cause of persistent airway obstruction. Despite offering five electrode configuration options, sixteen patients experienced a persistent lack of palatal coupling. No baseline parameters were identified that distinguished these patients from rest of the cohort.

For the patients in which upper airway stimulation alone seems not to be effective, additional therapy becomes an option when alternation of settings is insufficient or palatoglossal coupling is lacking. This additional therapy is generally well-tolerated and our results suggest it may lead to improved clinical outcomes for some.

This study was conducted as an exploratory analysis aimed at evaluating the potential role of the DISE in patients who do not respond adequately to upper airway stimulation therapy after other titration methods within the current clinical pathway have failed. Instead of testing a predefined hypothesis, we aimed to present our data to guide future research or clinical decision-making. We do realize that firm conclusions cannot be drawn with the limitations of a rather small patient sample size and the retrospective nature of this study. Additionally, we acknowledge more than half of the patients are unfortunately not effectively treated after the DISE with UAS. Therapy has mainly improved with the adjustments of device settings and the use of BRP. None of the patients who underwent OAT or uvulectomy were classified as a responder after additional treatment.

Nevertheless, it is noteworthy that five out of six patients who underwent adjustments in device settings were responders at follow-up. Even though these patients underwent the standard titration steps, including awake titration and titration PSG, DISE provided new insights for optimal device settings. The DISE procedure, being the golden standard for assessing upper airway collapse and can be used as a valid model of natural sleep [13], may offer advantages in evaluating nocturnal airway collapse patterns and the effectiveness of upper airway stimulation in some patients during sleep.

Therefore, this procedure can be a valuable addition for this specific group of patients who have not benefited from previous treatment attempts and are currently facing difficulties with their last therapy option. This is to say, that only after no adequate treatment results have been achieved after awake titration or titration PSG, a DISE with UAS could be recommended. When considering this procedure, it is important to weigh the potential benefits, both in terms of symptom improvement and long-term impact of OSA on the patient’s health, against the costs, including financial expenses and potential side effects or risks associated with this procedure.

Patients who are potential candidates for a DISE with UAS are those that are non-responders, but demonstrate an acceptable tolerance for therapy. Awake titration is more suitable for patients facing problems regarding tolerance, as it allows for adjustments regarding comfort settings while the patient is responsive.

Currently, there is no standardized procedure for DISE with UAS in the clinical pathway. Our study does not directly evaluate the usefulness of the DISE, but outlines our procedure, emphasizing a thorough check of all device settings and its effect on the upper airway. This article aims to provide an overview of our procedure and our patient cohort, establishing a foundation for further research to formally assess the effectiveness of this procedure and its potential inclusion in the standard clinical pathway of UAS therapy.

## Conclusion

The drug-induced sleep endoscopy might be a valuable addition in the clinical pathway of upper airway stimulation therapy, as it may give benefit to patients who have not responded to previous titration interventions. It can provide a better understanding of the problem and the possibility to change device settings or assess for additional therapy to improve upper airway stimulation therapy. Further research is necessary to determine the viability of this procedure and its potential inclusion in the clinical pathway.

## Data Availability

Data will be made available on reasonable request.
